# Relationship of low molecular weight fluorophore levels with clinical factors and fenofibrate effects in adults with type 2 diabetes

**DOI:** 10.1038/s41598-021-98064-y

**Published:** 2021-09-21

**Authors:** Andrzej S. Januszewski, David Chen, Russell S. Scott, Rachel L. O’Connell, Nanda R. Aryal, David R. Sullivan, Gerald F. Watts, Marja-Riitta Taskinen, Philip J. Barter, James D. Best, R. John Simes, Anthony C. Keech, Alicia J. Jenkins

**Affiliations:** 1 National Health and Medical Research Council Clinical Trials Centre, The University of Sydney, Level 6 Medical Foundation Building, 92-94 Parramatta Rd, Camperdown, Sydney, NSW 2050 Australia; 2 Monash School of Medicine, Monash University, Melbourne, VIC Australia; 3 Lipid and Diabetes Research Group, Christchurch Hospital, Christchurch, New Zealand; 4 Royal Prince Alfred Hospital, Sydney, NSW Australia; 5 Faculty of Health and Medical Sciences, School of Medicine, University of Western Australia, Perth, Australia; 6 Lipid Disorders Clinic, Cardiometabolic Services, Department of Cardiology, Royal Perth Hospital, Perth, Australia; 7 Cardiovascular Research Unit, Helsinki, Heart and Lung Centre, University Central Hospital, Helsinki, Finland; 8 Diabetes and Obesity Research Program, University of Helsinki, Helsinki, Finland; 9 Centre for Vascular Research, University of New South Wales, Sydney, NSW Australia; 10 Faculty of Medicine, The University of Sydney, Sydney, NSW Australia; 11 Lee Kong Chian School of Medicine, Nanyang Technical University, Singapore, Singapore; 12 Department of Medicine, St Vincent’s Hospital, University of Melbourne, Melbourne, VIC Australia

**Keywords:** Endocrinology, Diabetes

## Abstract

People with diabetes are at risk of chronic complications and novel biomarkers, such as Advanced glycation end-products (AGEs) may help stratify this risk. We assessed whether plasma low-molecular weight AGEs, also known as LMW-fluorophores (LMW-F), are associated with risk factors, predict complications, and are altered by fenofibrate in adults with type 2 diabetes. Plasma LMW-F were quantified at baseline, after six weeks fenofibrate, and one year post-randomisation to fenofibrate or placebo. LMW-F associations with existing and new composite vascular complications were determined, and effects of fenofibrate assessed. LMW-F correlated positively with age, glycated haemoglobin (HbA1c), pulse pressure, kidney dysfunction and inflammation; and negatively with urate, body mass index, oxidative stress and leptin, albeit weakly (r = 0.04–0.16, all *p* < 0.01). Independent determinants of LMW-F included smoking, diastolic blood pressure, prior cardiovascular disease or microvascular complications, Caucasian ethnicity, kidney function, HbA1c and diabetes duration (all *p* ≤ 0.01). Baseline LMW-F tertiles correlated with on-trial macrovascular and microvascular complications (trend *p* < 0.001) on univariate analyses only. Six weeks of fenofibrate increased LMW-F levels by 21% (*p* < 0.001). In conclusion, LMW-F levels correlate with many risk factors and chronic diabetes complications, and are increased with fenofibrate. LMW-F tertiles predict complications, but not independently of traditional risk factors.

## Introduction

Advanced glycation end-products (AGEs) are a complex, heterogenous group of fluorescent and non-fluorescent compounds formed through non-enzymatic reactions between reducing sugars, proteins, lipids or nucleic acids, and are implicated in the pathogenesis of vascular damage^1^. AGEs may be both a cause and effect of inflammation, oxidative stress and dyslipoproteinaemia^2^. In diabetes, AGEs accumulate in sites of complications including the kidney, retina and arteries^3–5^. Experimental studies show that some AGE-modulating drugs (e.g., aminoguanidine and ACE inhibitors) ameliorate diabetes complications^6,7^.

Low-molecular weight (< 10 kDa) AGEs (also known as LMW-AGEs, AGE peptides or LMW-fluorophores (LMW-F)) form via the incomplete degradation of AGE-modified proteins^8^. It is hypothesised that LMW-AGEs may be more toxic than larger AGEs via interactions with more distant tissue receptors via the circulation^9^. AGE degradation may facilitate renal excretion^10^ and, in keeping, serum AGE peptides and urinary AGE peptide excretion rates have been correlated^11^. Quantification of specific AGEs often requires sophisticated research assays, but LMW-AGEs can be measured by simple non-specific fluorescence spectroscopy^12,13^. As this technique is flourescence-based and not all AGEs fluoresce, we refer to the biomarker herein as LMW-F. While their chemical composition is unknown, levels have been correlated with serum AGEs (by ELISA)^13^ and with tissue auto-fluorescence^14^. Serum LMW-F levels can be lowered by aminoguanidine^14^ and circulating levels are regarded as an indicator of tissue AGEs^14,15^.

In a previous longitudinal study, we demonstrated higher plasma LMW-F levels in people with type 1 diabetic kidney damage, and correlations with measures of inflammation, oxidation and vascular dysfunction^16^. However, there are limited data on associations of LMW-Fs with a range of diabetes complications and other novel biomarkers, and there are no data on fenofibrate effects. While the primary coronary heart disease endpoint of the Fenofibrate Intervention and Event Lowering in Diabetes (FIELD) trial in 9795 adults with type 2 diabetes was negative, fenofibrate significantly reduced some macrovascular events, sight-threatening diabetic retinopathy, kidney damage and amputations^17–21^. The size, design, event numbers and duration of the FIELD trial provide a unique, robust, time and cost-effective opportunity to assess the potential utility of novel biomarkers such as LMW-F. This study assessed whether plasma LMW-F levels at baseline: (i) are associated with traditional and novel vascular risk factors; (ii) predict chronic complications; and (iii) are altered by fenofibrate in adults with type 2 diabetes.

## Materials and methods

### Study design

The study design and major FIELD trial results have already been published^17–21^. Briefly, the FIELD study was a double-blind, placebo-controlled, randomised trial with 9795 50–75-year-old participants with type 2 diabetes (International Standard Randomised Controlled Trial no. ISRCTN64783481). All subjects received dietary advice, single-blinded placebo, then six weeks once-daily 200 mg co-micronised fenofibrate pre-randomisation to placebo or fenofibrate for a median of five years. The study was approved by the University of Sydney Human Research Ethics Committee (#2012/402), and undertaken in accordance with the Declaration of Helsinki and Good Clinical Practice guidelines. All participants provided written informed consent. LMW-F levels were measured at baseline and at Visit 4 (16 weeks post-baseline), and a randomly selected subset of 1994 subjects also had Visit 7 (1 year) levels quantified.

### Vascular events

Details of all on-study vascular events are published elsewhere^17,18^. Briefly, (composite) macrovascular events included myocardial infarction, stroke, cardiovascular death and coronary or carotid revascularisation. Incident (composite) microvascular events were peripheral neuropathy (abnormal monofilament test); nephropathy (urinary albumin-to-creatinine ratio (ACR) ≥ 2.5 mg/mmol for men and ≥ 3.5 mg/mmol for women); retinopathy (on-study retinal laser for diabetic retinopathy); and major or minor amputation without known peripheral vascular disease in the same limb.

### Biomarker measurement

Venous blood after an overnight fast and a single void urine sample were collected. For research tests, plasma and serum were stored (− 80 °C) until analysis (see below).

LMW-F measurement has been previously described^12,13,16,22^. Intra- and inter-assay coefficients of variation (CV) were 3.8% and 4.1%, respectively, and the mean of two replicates was used in data analyses. All samples for the same participant were analysed in the same run. In preliminary studies (not shown), we demonstrated lack of intrinsic fluorescence of fenofibrate (Sigma-Aldrich St. Louis, MO, USA).

Oxidised low-density lipoprotein (Ox-LDL) and myeloperoxidase (MPO) were measured by ELISA (R&D Systems, Inc., Minneapolis, MN). Soluble vascular cell adhesion molecule-1 (sVCAM-1), soluble intercellular adhesion molecule (sICAM), soluble E-selectin (sE-Selectin), and interleukin-6 (IL-6) were all measured by ELISA (R&D Systems, Inc., Minneapolis, MN). Levels of the pro-inflammatory, pro-oxidant adipokine leptin were by determined ELISA (R&D Systems, Inc., Minneapolis, MN). All intra- and inter-assay CVs were < 10%.

### Statistics

Analyses were on an intention-to-treat basis. Comparisons of baseline characteristics across ordered groups used Mantel–Haenszel ordered tests for categorical variables, and linear trend tests (via simple linear regression) for continuous variables. Two-group comparisons used χ^2^-tests for categorical variables and t-tests for continuous variables. Baseline adjusted mean LMW-F levels and p-values were via multiple linear regression for comparisons according to history of chronic complications. Non-normally distributed continuous variables were natural log-transformed and geometric means and standard deviation presented. Spearman’s rank-order correlation was calculated to measure strength and direction of associations between continuous variables.

Relationships between variables and LMW-F levels at baseline were assessed using univariable and multivariable linear regression with log-transformed LMW-F as the dependent variable. All variables with *p* < 0.20 in univariate analyses were included in an exhaustive search procedure to select variables for inclusion in the final multivariable model. Logistic regression was used to identify independent predictors of LMW-F change during active run-in with fenofibrate. Cox regression was used to assess associations of baseline LMW-F tertiles with occurrence of cardiovascular events, new retinopathy and amputation over (median 5 years) follow-up.

Biomarker values below the lower limit of detection were imputed as half the next smallest value. All results are reported on original scales. Statistical inferences were drawn with a two-sided p-value of 0.05. Results are presented unadjusted for multiple comparisons. SAS (version 9.4; SAS Institute Inc., Cary, NC, USA) and SPSS (SPSS Inc, Chicago IL) software were used.

## Results

### Baseline characteristics

Baseline characteristics are given in Table 1 for all participants with LMW-F values (n = 9769) and as divided by LMW-F tertiles. LMW-F levels were unavailable for 26 of 9795 FIELD participants.

**Table 1:** Subject demographics and biomarker (traditional and novel) levels at baseline including by LMW-F tertiles.

	Overall	LMW-F	LMW-F	LMW-F	*p* value*
	(n = 9769)	Tertile 1 (< 2.74) (n = 3256)	Tertile 2 (2.74 to 3.72) (n = 3257)	Tertile 3 (> 3.72) (n = 3256)	
**General characteristics**					
Age at Visit 1 (years)	62.2 ± 6.9	61.4 ± 6.8	62.0 ± 6.9	63.3 ± 6.8	< **0.001**
Male	6123 (63%)	1970 (61%)	2068 (64%)	2085 (64%)	**0.003**
Caucasian	9068 (93%)	2974 (91%)	3029 (93%)	3065 (94%)	< **0.001**
Diabetes duration^†^ (years)	4.3 (2.8)	4.0 (2.8)	4.2 (2.9)	4.8 (2.8)	< **0.001**
HbA1c^†^ (mmol/mol)	51.8 (1.3)	50.9 (1.3)	52.0 (1.3)	52.4 (1.3)	< **0.001**
HbA1c^†^ (%)	6.94 (1.20)	6.86 (1.20)	6.96 (1.20)	7.00 (1.20)	< **0.001**
HOMA2-IR^†^	1.8 (1.8)	1.8 (1.8)	1.7 (1.8)	1.8 (1.8)	0.36
BMI^†^ (kg/m^2^)	30.2 (1.2)	30.7 (1.2)	30.0 (1.2)	29.9 (1.2)	< **0.001**
Waist-to-hip ratio^†^	0.9 (1.1)	0.9 (1.1)	0.9 (1.1)	0.9 (1.1)	0.66
Systolic BP (mmHg)	140 ± 15	140 ± 15	141 ± 15	141 ± 16	0.19
Diastolic BP (mmHg)	82 ± 9	83 ± 8	82 ± 9	81 ± 9	< **0.001**
Pulse pressure (mmHg)	58 ± 12	58 ± 12	58 ± 13	59 ± 13	< **0.001**
Current smoker	921 (9%)	228 (7%)	312 (10%)	381 (12%)	< **0.001**
**Clinical history**					
Prior CVD	2124 (22%)	567 (17%)	684 (21%)	873 (27%)	< **0.001**
Myocardial infarction	483 (5%)	122 (4%)	153 (5%)	208 (6%)	< **0.001**
Stroke	346 (4%)	80 (3%)	119 (4%)	147 (5%)	< **0.001**
Angina	1181 (12%)	291 (9%)	395 (12%)	495 (15%)	< **0.001**
Peripheral vascular disease	763 (8%)	225 (7%)	214 (7%)	324 (10%)	< **0.001**
Coronary revascularisation (CABG or PTCA)	362 (4%)	101 (3%)	111 (3%)	150 (5%)	**0.001**
History of hypertension	5528 (57%)	1783 (57%)	1795 (55%)	1950 (60%)	< **0.001**
Microvascular disease	3262 (33%)	994 (31%)	1029 (32%)	1239 (38%)	< **0.001**
Retinopathy	814 (8%)	237 (7%)	258 (8%)	319 (10%)	< **0.001**
Neuropathy	558 (6%)	140 (4%)	175 (5%)	243 (8%)	< **0.001**
Nephropathy	2501 (26%)	784 (24%)	785 (24%)	932 (29%)	< **0.001**
**Renal function**					
Plasma creatinine (μmol/L)	77.6 ± 15.8	75.0 ± 15.2	78.0 ± 15.6	79.8 ± 16.2	< **0.001**
eGFR (mL/min/1.73 m^2^)	84.5 ± 14.1	87.1 ± 13.5	84.4 ± 13.8	82.1 ± 14.7	< **0.001**
Urine albumin-creatinine ratio^†^ (mg/mmol)	1.5 (3.7)	1.4 (3.6)	1.5 (3.5)	1.7 (4.0)	< **0.001**
Cystatin C^†^ (mg/L)	0.9 (0.2)	0.9 (0.2)	0.9 (0.2)	1.0 (0.2)	< **0.001**
Uric acid^†^ (mmol/L)	0.32 (1.27)	0.33 (1.26)	0.33 (1.26)	0.31 (1.28)	< **0.001**
**Lipids**					
Total cholesterol (mmol/L)	5.0 ± 0.7	5.1 ± 0.7	5.0 ± 0.7	5.0 ± 0.7	0.14
HDL-cholesterol (mmol/L)	1.10 ± 0.26	1.10 ± 0.26	1.10 ± 0.26	1.08 ± 0.26	**0.04**
LDL-cholesterol (mmol/L)	3.1 ± 0.7	3.1 ± 0.6	3.1 ± 0.6	3.1 ± 0.7	0.59
Triglycerides^†^ (mmol/L)	1.78 (1.50)	1.81 (1.51)	1.77 (1.48)	1.77 (1.50)	**0.01**
**Baseline glucose-lowering medication**					
Diet alone	2602 (27%)	885 (27%)	905 (28%)	812 (25%)	< **0.001**
Oral agent alone	5823 (60%)	2007 (62%)	1883 (58%)	1933 (59%)	
Insulin alone	605 (6%)	168 (5%)	192 (6%)	245 (8%)	
Insulin + oral agent	739 (8%)	196 (6%)	277 (9%)	266 (8%)	
**Novel biomarkers**					
Homocysteine^†^ (V3 only) (μmol/L)	9.7 (1.3)	9.4 (1.3)	9.7 (1.3)	10.0 (1.4)	< **0.001**
**Inflammation**					
White cell count^†¶^ (× 10^9^/L)	6.6 (1.3)	6.6 (1.3)	6.5 (1.3)	6.7 (1.3)	**0.02**
hs-CRP^†^ (mg/L)	2.8 (3.0)	2.9 (3.0)	2.5 (3.0)	2.9 (3.0)	0.42
sVCAM-1^†^ (ng/mL)	633.7 (1.4)	641.1 (1.4)	617.4 (1.4)	642.7 (1.5)	0.45
sICAM^†¶^ (ng/mL)	249.7 (1.3)	248.4 (1.3)	245.7 (1.3)	255.2 (1.3)	< **0.001**
sE-selectin^†‡^ (ng/mL)	32.6 (1.6)	33.7 (1.6)	32.2 (1.6)	31.8 (1.6)	< **0.001**
IL-6^†‡¶^ (pg/mL)	2.5 (1.9)	2.5 (1.8)	2.4 (1.9)	2.7 (1.9)	< **0.001**
Fibrinogen (g/L)	3.6 ± 0.7	3.6 ± 0.7	3.6 ± 0.7	3.6 ± 0.8	**0.008**
**Oxidative stress**					
Myeloperoxidase^†‡^ (μg/L)	50.7 (1.9)	50.8 (1.8)	47.5 (1.9)	54.1 (2.0)	< **0.001**
ox-LDL^†^ (mU/L)	39.7 (1.6)	41.4 (1.6)	39.3 (1.6)	38.5 (1.6)	< **0.001**
ox-LDL/LDL^†^ (mU/mmol)	13.3 (1.6)	13.9 (1.6)	13.1 (1.7)	12.9 (1.7)	< **0.001**
**Adipokines**					
Leptin^†‡¶^ (pg/mL)	8281 (3)	8971 (3)	7925 (3)	7989 (3)	< **0.001**

*p* values <  0.05 are bolded

*CABG* Coronary artery bypass grafting, *PTCA* Percutaneous transluminal coronary angioplasty.

*Variables were compared across the three tertiles using the Mantel–Haenszel ordered test for categorical variables and linear trend for continuous variables.

^†^Log-transformed, geometric means and geometric SD factors presented.

^‡^The values below the lower limit of detection were imputed as half the smallest value.

^¶^Overall relationship for these variables was quadratic with significant linear and quadratic components.

Supplementary Fig. 1 shows the non-normally distributed plasma LMW-F levels at baseline, with a median of 3.21 AU (IQR = 2.49–4.06 AU, range = 0–137.96 AU). There was no significant difference in baseline LMW-F by sex or subsequent treatment group. Smoking was associated with higher LMW-F levels, with current smokers having 5.2% and 9.7% higher LMW-F levels than ex-smokers and subjects who had never smoked, respectively (geometric mean: current smokers 3.57 vs. ex-smokers 3.39 AU vs. never smoked 3.25 AU, trend *p* < 0.001).

As shown in Table 1, subjects in higher LMW-F tertiles were older, had longer known diabetes duration, higher HbA1c and pulse pressure, and lower body mass index (BMI), HDL-cholesterol, triglycerides, serum uric acid and worse kidney function, although many differences were small. They were more likely to be male, Caucasian, smokers, on insulin alone or have prior macro- or microvascular disease. Leptin and many biomarkers of inflammation and oxidative stress also differed.

### Associates of baseline LMW-F levels

Table 2 shows statistical determinants of baseline LMW-F levels on unadjusted analyses and in a multivariate linear regression model. In univariate analyses, LMW-F levels (continuous variable) were weakly associated with age, Caucasian ethnicity, diabetes duration, HbA1c, BMI (inverse), blood pressure, prior cardiovascular disease (CVD) and microvascular disease, smoking status, insulin use, uric acid (inverse) and kidney dysfunction (positive associations with plasma creatinine, cystatin C, urine albumin-creatinine ratio and homocysteine, and negative association with eGFR) (r = 0.02–0.16, all *p* ≤ 0.02).

**Table 2:** Relationship between baseline variables (standardised*) and LMW-F levels at baseline.

	Unadjusted		Percentage change (95% CI)	Exhaustive search		Percentage change (95% CI)
	B	*p*		B	*p*	
**General characteristics**						
Age	0.07	< **0.001**	7.1 6.0, 8.3)			
Male	− 0.001	0.97	− 0.1 (− 2.3, 2.2)			
Caucasian	0.11	< **0.001**	12.1 (7.4, 16.9)	0.09	< **0.001**	9.4 (4.9, 14.1)
Diabetes duration	0.04	< **0.001**	4.1 (3.0, 5.3)	0.02	**0.01**	1.5 (0.3, 2.7)
HbA1c	0.01	**0.03**	1.2 (0.1, 2.4)	− 0.02	**0.009**	− 1.6 (− 2.8, − 0.4)
HOMA2-IR	0.008	0.18	0.8 (− 0.4, 1.9)	0.02	**0.002**	1.8 (0.6, 2.9)
BMI	− 0.02	**0.003**	− 1.7 (− 2.7, − 0.6)			
Waist-to-hip ratio	− 0.006	0.33	− 0.6 (− 1.6, 0.5)			
Systolic BP	0.01	**0.03**	1.3 (0.2, 2.4)			
Diastolic BP	− 0.03	< **0.001**	− 3.0 (− 4.1, − 2.0)	− 0.02	< **0.001**	− 2.0 (− 3.1, − 0.9)
Prior CVD	0.15	< **0.001**	16.7 (13.6, 19.8)	0.10	< **0.001**	10.4 (7.4, 13.5)
Prior microvascular disease	0.08	< **0.001**	8.6 (6.1, 11.2)	0.06	< **0.001**	6.1 (3.5, 8.7)
**Smoking**						
Current smoker	0.09	< **0.001**	9.7 (5.4, 14.1)	0.11	< **0.001**	11.2 (6.8, 15.8)
Ex-smoker	0.04	< **0.001**	4.2 (1.9, 6.7)	0.05	< **0.001**	5.3 (2.9, 7.7)
**Baseline glucose-lowering medications**						
Oral agent alone	0.02	0.21	1.6 (− 0.9, 4.3)			
Insulin alone	0.09	< **0.001**	9.3 (4.1, 14.8)			
Insulin + oral agent	0.08	< **0.001**	7.9 (3.1, 12.9)			
**Renal function**						
Plasma creatinine	0.07	< **0.001**	7.4 (6.2, 8.5)			
eGFR	− 0.09	< **0.001**	− 8.9 (− 9.9, − 7.9)	− 0.10	< **0.001**	− 9.2 (− 10.5, − 7.9)
Urine albumin-creatinine ratio	0.03	< **0.001**	2.5 (1.4, 3.7)			
Cystatin C	0.06	< **0.001**	6.4 (5.2, 7.6)	0.02	**0.005**	2.1 (0.6, 3.6)
Uric acid	− 0.05	< **0.001**	− 4.5 (− 5.5, 3.4)	− 0.09	< **0.001**	− 8.2 (− 9.3, − 7.1)
**Lipids**						
Total cholesterol	− 0.005	0.34	− 0.5 (− 1.6, 0.6)			
HDL-cholesterol	− 0.005	0.35	− 0.5 (− 1.6, 0.6)			
LDL-cholesterol	− 0.003	0.61	− 0.3 (− 1.4, 0.8)			
Triglycerides	− 0.004	0.51	− 0.4 (− 1.5, 0.7)			
**Novel biomarkers**						
hs-CRP	0.009	0.10	0.9 (− 0.2, 2.1)			
Fibrinogen	0.01	**0.03**	1.3 (0.1, 2.4)			
Homocysteine	0.04	< **0.001**	4.1 (3.0, 5.3)			

*p* values <  0.05 are bolded

*Continuous variables have been standardised such that results relate to a 1 SD change.

Due to high correlation between plasma creatinine and eGFR (r =  − 0.80), only eGFR was included in multivariable analysis.

LMW-F levels also correlated weakly with inflammation (sICAM, sE-selectin, IL-6, fibrinogen), oxidative stress (ox-LDL, ox-LDL/LDL) and leptin (r = 0.03–0.08, all *p* < 0.01), but was not correlated with the clinically available measures of white cell count and CRP (both *p* > 0.05).

In the multivariate model, LMW-F determinants were smoking, prior cardiovascular disease (CVD), Caucasian ethnicity, eGFR, uric acid, prior microvascular disease, cystatin C, diastolic BP, HOMA2-IR, HbA1c and diabetes duration (all *p* ≤ 0.01). This model explained 7% of LMW-F variability.

### Associations of baseline LMW-F levels with baseline macrovascular complications

As shown (Supplementary Table 1), participants with macrovascular complication(s) at baseline had 17% higher LMW-F levels (*p* < 0.001) versus those without. After adjustment for confounding variables, LMW-F levels remained significantly higher in all conditions (myocardial infarction, stroke, angina, peripheral vascular disease) except coronary revascularisation.

### Associations of baseline LMW-F levels with baseline microvascular complications

Participants with (composite) microvascular disease at baseline had 9% higher LMW-F levels (*p* < 0.001) versus those without (Supplementary Table 2). Without adjustment, of the four individual components of the composite endpoint, LMW-F levels differed between subjects with and without nephropathy (*p* < 0.001), neuropathy (*p* < 0.001) and retinopathy (*p* = 0.01), but not by microvascular amputation status (*p* = 0.87). However, after adjustment for confounding variables, LMW-F levels remained significantly higher only between subjects with versus without composite microvascular disease and neuropathy (both *p* < 0.001).

### Associations of baseline LMW-F tertiles with new on-study macrovascular complications

**Figure 1:**
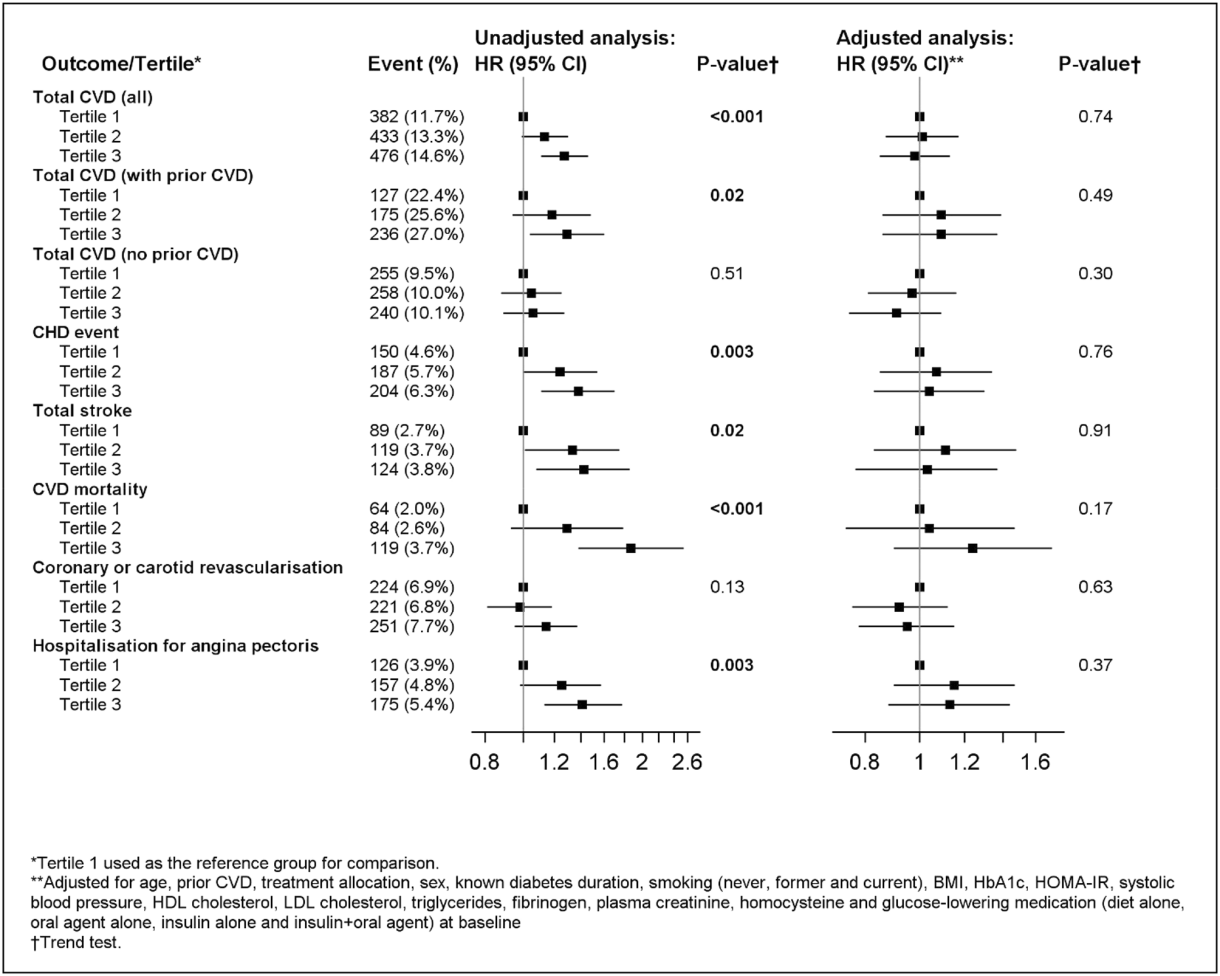
Association of baseline LMW-F levels with on-study CVD events over 5 years.

During the (median) five-year follow-up, 1291 patients (13.2%) had ≥ 1 macrovascular event. Figure 1 shows associations of baseline LMW-F tertiles with on-trial macrovascular events. Without adjustment, higher LMW-F tertiles were associated with higher hazard ratios for total CVD events, CHD events, stroke, CVD mortality and hospitalisation for angina (all overall effect *p* ≤ 0.02), driven mainly by effects in the prior CVD subgroup. After adjustment for confounders, baseline LMW-F tertiles were no longer independently associated with on-trial CVD. Variables attributing most to this loss of association were age, prior CVD and plasma creatinine (Supplementary Table 3).

### Associations of baseline LMW-F tertiles with new on-study microvascular complications

**Figure 2:**
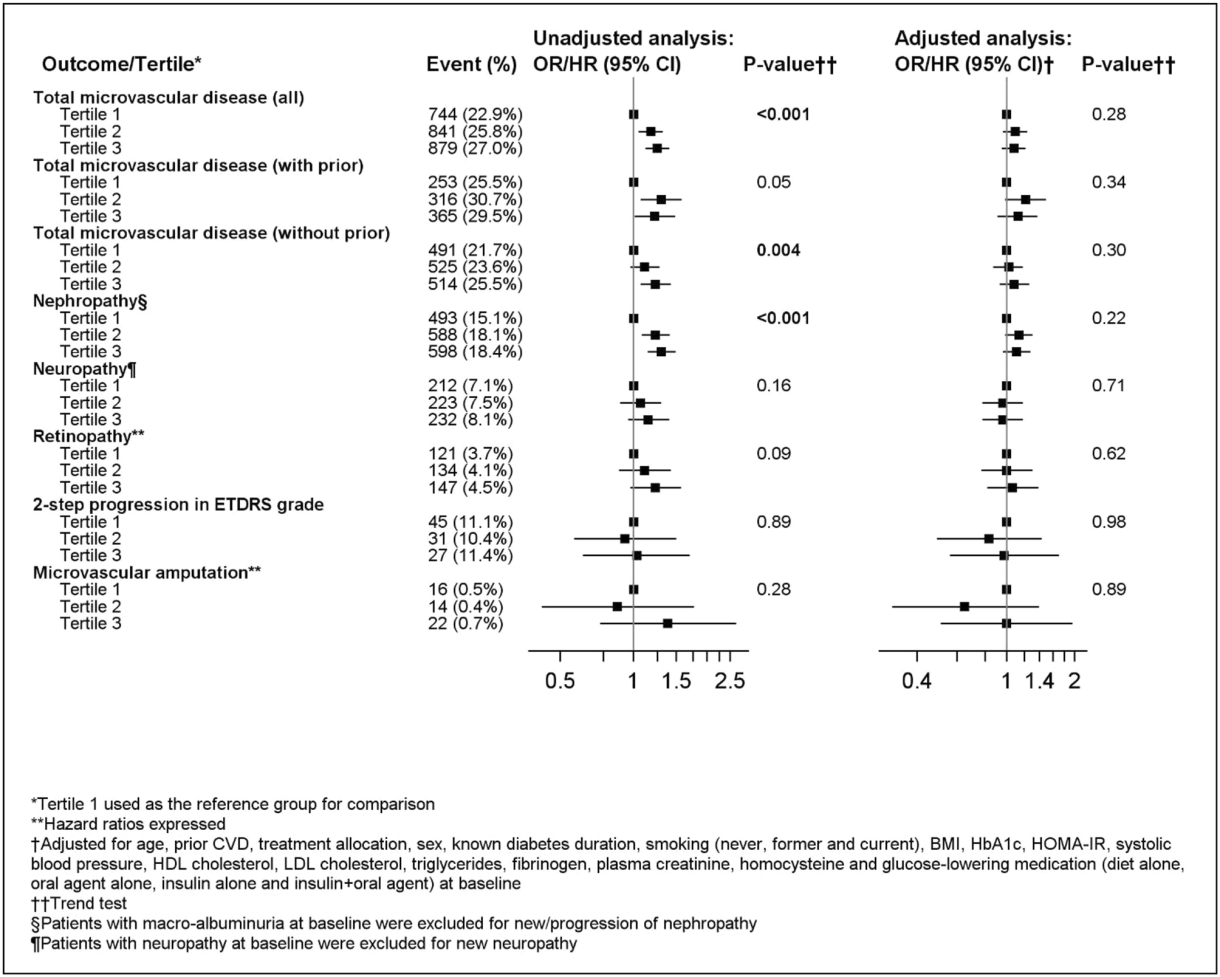
Association of baseline LMW-F levels with on-study microvascular events over 5 years.

During the (median) five-year follow-up, 2464 participants (25.2%) had ≥ 1 new microvascular event or microvascular disease progression. New or worsening nephropathy (1679, or 17.2% of the cohort) and new neuropathy (667, or 6.8% of the cohort) were the most common microvascular complications. Figure 2 shows associations of baseline LMW-F tertiles with microvascular events. Without adjustment, higher LMW-F tertiles were associated with higher hazards for total microvascular disease and nephropathy (both *p* ≤ 0.001), which was stronger in participants without prior microvascular disease. However, significance was lost after adjustment for confounding variables. Age, prior CVD, diabetes duration, smoking, treatment allocation, HbA1c, serum homocysteine and systolic BP contributed to this loss (Supplementary Table 4).

### Changes in LMW-F levels

**Figure 3:**
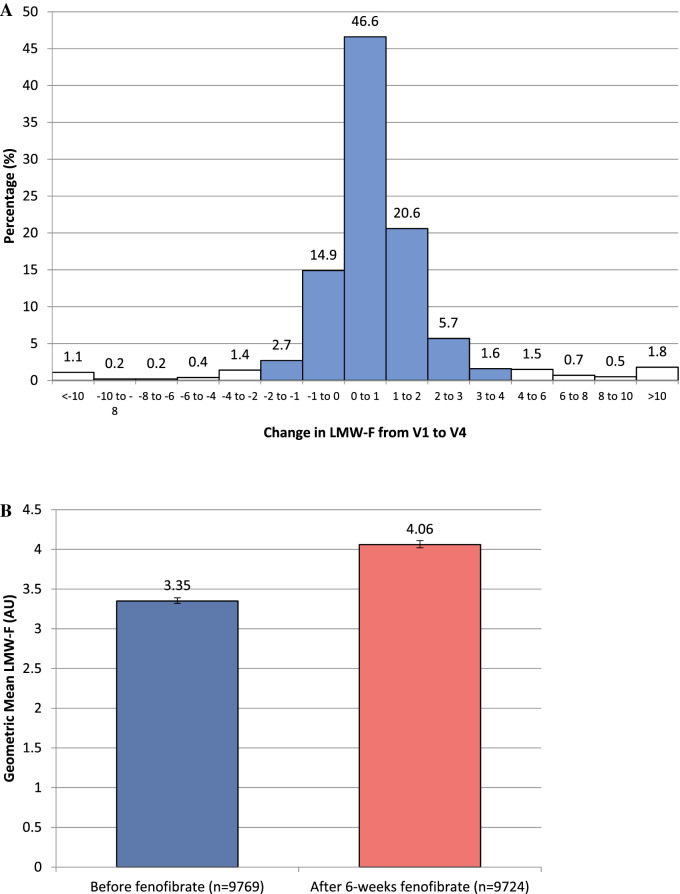

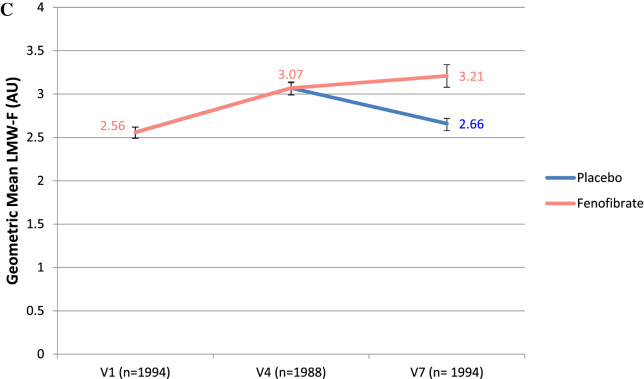
(**A**) Distribution of change in LMW-F between V1 and V4 16 weeks apart and after 6-weeks fenofibrate. (**B**) LMW-F levels before and after 6-weeks fenofibrate (*p* < 0.001). (** C**) Changes in LMW-F levels over time and with fenofibrate in participants with LMW-F measurements at V7. All participants received fenofibrate from V1 to V4 (for the last 6 weeks), before being subsequently randomised to 5 years of fenofibrate or placebo. Error bars show 95% CIs.

Figure 3 shows plasma LMW-F level changes over time by on-trial fenofibrate or placebo allocation. Individual responses varied widely (Fig. 3, Panel A). LMW-F levels increased in 79% of subjects. From baseline to Visit 4, which included single-blind fenofibrate for the last six weeks in all participants immediately pre-randomisation, LMW-F levels increased by 21% (Fig. 3, Panel B). Supplementary Table 5 shows determinants of LMW-F change during run-in. In the multivariate model, higher systolic BP and serum uric acid increased the odds of LMW-F increasing, while higher baseline LMW-F, HOMA2-IR, urine albumin-creatinine ratio, sVCAM-1 and myeloperoxidase decreased the odds. The baseline LMW-F level significantly influenced the response to six weeks of fenofibrate treatment in both univariate and exhaustive search analyses (Supplementary Table 5). Of participants whose LMW-F decreased following six weeks of fenofibrate, 57% were in the highest baseline LMW-F tertile and 18% were in the lowest tertile.

In the randomly selected 1994 participants also evaluated at one year (Visit 7) to determine durability of changes with fenofibrate (Fig. 3, Panel C), six weeks of fenofibrate (Visit 4) increased LMW-F levels by 20% relative to baseline, with similar changes in the groups by subsequent randomisation to long-term fenofibrate or placebo. From Visit 4 to 7, LMW-F levels increased by 3% with fenofibrate, and decreased towards baseline with placebo. As shown (Fig. 3, Panels B and C), LMW-F levels were significantly higher at baseline and Visit 4 in participants with two LMW-F measurements (V1 and V4) than with three measures (V1, V4 and V7), in spite of clinically minimal differences in most baseline characteristics (Supplementary Table 6).

### Correlates of LMW-F change during the six-week active run-in

Changes in LMW-F levels from baseline to Visit 4 were associated, albeit weakly, with changes in systolic blood pressure, kidney function (plasma creatinine, eGFR, cystatin C), serum uric acid, HDL-cholesterol, triglycerides, sICAM, myeloperoxidase and leptin (all *p* ≤ 0.02) (Supplementary Table 5). In a multivariate linear regression model (Table 3), independent predictors of LMW-F change were change in serum uric acid, plasma creatinine, cystatin C, MPO, HDL-cholesterol and systolic blood pressure (all *p* ≤ 0.02).

**Table 3:** Relationship change in baseline variables between V1 and V4 (16 weeks apart and at the end of 6 weeks of fenofibrate) (standardised*) and change in LMW-F between V1 and V4 (log-transformed).

	Unadjusted		Percentage change (95% CI)	Exhaustive search		Percentage change (95% CI)
	B	*p*		B	*p*	
**General characteristics**						
Δ BMI	− 0.007	0.11	− 0.7 (− 1.5, 0.1)			
Δ Systolic BP	− 0.01	**0.001**	− 1.3 (− 2.1, − 0.5)	− 0.01	**0.02**	− 1.0 (− 1.8, − 0.2)
Δ Diastolic BP	− 0.008	0.05	− 0.8 (− 1.6, 0.0)			
**Renal function**						
Δ Plasma creatinine	0.05	< **0.001**	4.8 (4.0, 5.7)	0.04	< **0.001**	4.2 (3.3, 5.2)
Δ eGFR	− 0.04	< **0.001**	− 4.3 (− 5.0, − 3.5)			
Δ Cystatin C	0.03	< **0.001**	2.9 (2.1, 3.8)	0.02	< **0.001**	2.1 (1.1, 3.1)
Δ Uric acid	− 0.06	< **0.001**	− 5.3 (− 6.1, − 4.5)	− 0.06	< **0.001**	− 6.1 (− 6.8, − 5.3)
**Lipids**						
Δ Total cholesterol	− 0.0001	0.98	− 0.0 (− 0.8, 0.8)			
Δ HDL-cholesterol	0.01	< **0.001**	1.4 (0.6, 2.3)	0.01	**0.004**	1.2 (0.4, 2.1)
Δ LDL-cholesterol	0.001	0.82	0.1 (− 0.7, 0.9)			
Δ Triglycerides	− 0.01	**0.01**	− 1.1 (− 1.9, 0.3)			
**Novel biomarkers**						
Δ Hs-CRP	− 0.003	0.47	− 0.3 (− 1.1, 0.5)			
Δ sVCAM-1	0.004	0.40	0.4 (− 0.5, 1.2)			
Δ sICAM	0.01	**0.02**	1.0 (0.2, 1.8)			
Δ Se-selectin	0.004	0.34	0.4 (− 0.4, 1.2)			
Δ IL-6	0.006	0.17	0.6 (− 0.3, 1.4)			
Δ Fibrinogen	− 0.007	0.10	− 0.7 (− 1.5, 0.1)			
Δ Myeloperoxidase	0.01	< **0.001**	1.4 (0.6, 2.3)	0.02	< **0.001**	1.8 (0.8, 2.8)
Δ Ox-LDL	− 0.007	0.12	− 0.7 (− 1.5, 0.2)			
Δ Ox-LDL/LDL	− 0.007	0.12	− 0.7 (− 1.5, 0.2)			
Δ Leptin	0.01	**0.003**	1.2 (0.4, 2.1)			

*p* values <  0.05 are bolded

*Continuous variables have been standardised such that results relate to a 1 SD change.

### Treatment effect of fenofibrate versus placebo by tertiles of run-in LMW-F change

The effect of fenofibrate (vs. placebo) to reduce on-study CVD and microvascular events did not differ by tertiles of LMW-F change (Supplementary Table 7).

## Discussion

In well-characterised adults with type 2 diabetes, we quantified circulating LMW-F and its associations with vascular risk factors, chronic complications and response to fenofibrate. LMW-F levels correlated only weakly with traditional and novel risk factors and were associated with prior CVD and microvascular disease. Baseline LMW-F tertiles were significantly associated with on-study CVD and microvascular events, but not independently of traditional and novel risk factors. Fenofibrate significantly increased LMW-F levels, but such changes were not associated with CVD benefits of fenofibrate.

The observed associations of LMW-F levels are consistent with and expand the existing literature. Prior studies, the largest of which had 605 participants, demonstrated associations between LMW-AGEs and age^23,24^, diabetes duration^24^, HbA1c^16^, BMI^25^, blood pressure^23^, kidney dysfunction^22–24^ and HDL-cholesterol^26^. The strength of most associations was weaker in our study of over 9700, which we believe provides the most accurate representation given our very large sample size. The inverse weak relationship between LMW-F and serum uric acid is unexpected, as both LMW-F and uric acid levels increase with kidney dysfunction, though higher uric acid has antioxidant effects. While no traditional lipid parameters were associated with LMW-F, previous studies demonstrated correlations with total cholesterol, triglycerides and HDL-cholesterol^26,27^. However, we found that changes in LMW-F were associated with changes in HDL-cholesterol and triglycerides, albeit weakly.

AGEs may be both a cause and consequence of inflammation and oxidative stress^2^. We identified weak, but statistically significant, positive correlations between LMW-F and inflammation, and negative correlations with oxidative stress. The latter is unexpected. In our prior small study in type 1 diabetes, LMW-F were positively associated with inflammation and oxidative stress markers^16^. Given the very weak associations observed in FIELD, it is unlikely to be of major clinical significance. LMW-F also correlated negatively with leptin, an appetite suppressor produced by adipose tissue, which is in keeping with the inverse correlation found between LMW-F and BMI. Increased adipose tissue may trap AGEs, lowering circulating LMW-F levels^25^. Overall, LMW-F appeared to have complex associations with our comprehensive set of novel biomarkers. Further studies, such as with weight changes, are merited to determine the clinical significance of these findings.

The independent predictors of LMW-F in our multivariate regression model align with existing knowledge. AGEs are known to increase with longer diabetes duration^24^, smoking^28,29^, kidney dysfunction^16,22–24^ and are implicated in the pathogenesis of diabetes complications^6^. In a prior study (n = 604), haemoglobin levels were an independent predictor of LMW-AGEs (*p* = 0.001)^24^, although this was not found in our much larger study. The relationship between Caucasian ethnicity and LMW-F is of unknown significance, given that 93% of FIELD participants were Caucasian. The relationship between LMW-F and HbA1c and HOMA2-IR also appear to be in the opposite direction to that expected, although associations were relatively weak. Given that only 7% of the variability in LMW-F was explained by our model, there are likely to be other factors, perhaps genetic factors or total body AGE burden, which were not determined but are important in determining circulating LMW-F levels.

LMW-F levels were significantly higher in subjects with versus without baseline CVD or composite microvascular disease, which remained statistically significant even after adjusting for covariates. Neuropathy was the only individual microvascular complication associated with higher LMW-F levels following adjustment, which to our knowledge, is a novel finding in type 2 diabetes.

We present novel findings on the association of baseline LMW-F for future diabetes complications. Most previous LMW-F studies have been cross-sectional, and to our knowledge none have examined relationships between LMW-F levels and future events, as we have done. In our longitudinal analyses, tertiles of baseline LMW-F levels were significantly associated with both composite on-study CVD and microvascular disease, although these associations were not independent of a large number of covariates. For CVD events, this loss of association was mostly attributable to prior CVD, plasma creatinine and age. For microvascular complications, only new nephropathy was significantly associated with baseline LMW-F levels, with non-significant trends for neuropathy and retinopathy. Indeed, it is likely that the accumulation of LMW-F is both a cause and result of kidney dysfunction, a strong CVD risk factor. While previous studies have demonstrated increased LMW-F levels with concurrent kidney dysfunction^22–24^, we are the first to show that LMF-F tertiles are associated with future nephropathy. However, as all these associations weaken once risk factors are adjusted for, it is likely that LMW-F are mediators by which risk factors act and that LMW-F are not clinically useful for predicting complications. Overall, these findings in a very large cohort give some support for relationships between LMW-F, traditional and novel risk factors and chronic complications. These findings may have a greater role in understanding the pathogenesis of diabetes complications and the determinants of LMW-F rather than in clinical prognostication, although relatively large effects of fenofibrate on LMW-F levels were observed.

Interestingly, six weeks of fenofibrate increased LMW-F, which reversed at one year in participants randomised to placebo and persisted in those randomised to ongoing fenofibrate. Our study does not support strong links between the increase in LMW-F with fenofibrate and the drug’s clinical benefits. Fenofibrate is a PPARα agonist that reduces triglycerides via lipoprotein lipase activation^30^ and also has many pleiotropic effects^31^. Many AGEs are derived from lipoproteins^32^, although we are unaware of LMW-F arising from lipoprotein degradation. We found that increases in LMW-F were associated with improvements in some vascular risk factors. Changes in LMW-F were negatively associated with changes in systolic blood pressure, uric acid, triglycerides, and positively with HDL-C, albeit very weakly. LMW-F changes were also positively associated with changes in plasma creatinine, cystatin C, sICAM and myeloperoxidase and negatively with eGFR, although this may be a cause of LMW-F accumulation rather than the effect of fenofibrate-induced LMW-F changes, as inflammation and kidney dysfunction increase AGEs. An exhaustive search found that the strongest predictors of LMW-F change were changes in serum uric acid (negative), plasma creatinine, cystatin C, myeloperoxidase, HDL-cholesterol and systolic BP (negative). As shown in the FIELD trial, fenofibrate is renoprotective^33^ and the changes in plasma creatinine and eGFR do not represent a reduction in renal filtration capacity.

LMW-F changes following six weeks of fenofibrate varied amongst participants, although LMW-F increased in the vast majority. Higher systolic BP and uric acid increased the odds of LMW-F increasing, while higher LMW-F, HOMA2-IR, urine albumin-creatinine ratio, sVCAM-1 and MPO decreased the odds. Subjects in whom LMW-F decreased were much more likely to have higher LMW-F levels at baseline. In our subgroup analysis, participants who had moderate increases in LMW-F during run-in attained the greatest CVD benefit with fenofibrate, but there was no benefit for subjects from the lowest tertile of LMW-F change. While fenofibrate’s CVD benefits appear to be attenuated in people in the highest tertile of LMW-F change, this group of participants had worse renal function, which may have impaired their ability to excrete harmful AGE peptides. Hence relationships between LMW-F changes and clinical benefits of fenofibrate are not evident.

Fenofibrate is known to increase serum creatinine levels, without reductions in measured glomerular filtration rate, thought to be due to effects on proximal renal tubular handling of creatinine^33^. It is possible that fenofibrate has a similar effect on LMW-F renal excretion. Further investigation is merited. Urine LMW-F levels were not assessed herein due to lack of availability of urine samples but, in a previous study, plasma levels and urine LMW-F excretion rates correlated in diabetes^11^.

Study strengths included a robust design, large population size, high event numbers, detailed subject characterisation, long follow-up and treatment with fenofibrate or placebo. We addressed questions that generate new hypotheses on the role of LMW-F in diabetic complications and the effects of fenofibrate. A wide range of novel biomarkers was measured, including of inflammation and oxidative stress, which are related to AGEs. Furthermore, our LMW-F assay was inexpensive and easily replicable and so has potential to be used in other research. All time-points were measured in one run using duplicate assays to increase precision. Study limitations include the partial observational design of our study and unknown chemical nature of LMW-F. In addition, LMW-F were not measured in urine, nor in plasma at study close-out and wash-out, which might have provided additional data on the effects of fenofibrate.

In conclusion, this study provides new data describing LMW-F. We found that circulating LMW-F levels were weakly associated with many traditional and novel vascular risk factors and diabetes complications, and while there were divergent responses, on average LMW-F levels increased with fenofibrate. The level of increase was not strongly associated with the drug’s complication-related benefits. LMW-F may provide new insight into the pathogenesis of diabetes complications. Further studies are required on LMW-F to elucidate its chemical nature, role in the pathogenesis of diabetes complications and the response to fenofibrate and anti-diabetic therapies.

## Supplementary Information

Supplementary Information.

## Data Availability

Data availability will be considered upon request for projects with ethics approval.
