# Supplementary material for: Relationship of low molecular weight fluorophore levels with clinical factors and fenofibrate effects in adults with type 2 diabetes
Source: Sci Rep. 2021 Sep 21;11:18708. doi: 10.1038/s41598-021-98064-y (PMC8455555; doi:10.1038/s41598-021-98064-y)

Low-molecular weight fluorophores in Type 2 diabetes: Associations with baseline and on-study diabetes complications, and effects of fenofibrate in the Fenofibrate Intervention and Event Lowering in Diabetes (FIELD) Study

Andrzej S Januszewski, David Chen, Russell S Scott, Rachel L O'Connell, Nanda R Aryal, David R Sullivan, Gerald F Watts, M-R Taskinen, Philip J Barter, James D Best, R John Simes, Anthony C Keech Alicia J Jenkins, on behalf of the FIELD Study Investigators

Supplementary Table 1: Baseline LMW-F levels according to prior (self-reported) history of CVD

	n	Unadjusted Geometric mean (95% CI)	p value	Adjusted* Geometric mean (95% CI)	p value
**Total CVD**					
No	7645	3.24 (3.20-3.28)	**<0.001**	3.42 (3.34-3.49)	**<0.001**
Yes	2124	3.78 (3.68-3.89)		3.75 (3.64-3.87)	
**MI**					
No	9286	3.33 (3.29-3.36)	**<0.001**	3.48 (3.41-3.56)	**0.007**
Yes	483	3.87 (3.65-4.10)		3.75 (3.55-3.95)	
**Stroke**					
No	9423	3.33 (3.30-3.37)	**<0.001**	3.48 (3.41-3.56)	**0.004**
Yes	346	3.88 (3.64-4.15)		3.82 (3.59-4.06)	
**Angina**					
No	8588	3.29 (3.25-3.32)	**<0.001**	3.45 (3.38-3.53)	**<0.001**
Yes	1181	3.86 (3.72-4.00)		3.80 (3.66-3.94)	
**PVD**					
No	9059	3.32 (3.28-3.36)	**<0.001**	3.47 (3.40-3.55)	**0.001**
Yes	710	3.78 (3.58-3.98)		3.73 (3.57-3.90)	
**Coronary revascularisation (CABG or PTCA)**					
No	9407	3.34 (3.30-3.38)	**0.001**	3.49 (3.42-3.57)	0.26
Yes	362	3.67 (3.46-3.89)		3.62 (3.40-3.85)	

Geometric means (95% CI) have been reported

*In the adjusted model, geometric means and p values were adjusted for age, sex, treatment allocation, known diabetes duration, smoking (never, former and current), BMI, HbA_{1c}, HOMA-IR, systolic blood pressure, HDL-cholesterol, LDL-cholesterol, triglycerides, fibrinogen, plasma creatinine, homocysteine and glucose-lowering medication (diet alone, oral agent alone, insulin alone and insulin+oral agent) at baseline

Supplementary Table 2: Baseline LMW-F levels according to prior (self-reported) history of microvascular disease

	n	Unadjusted Geometric mean (95% CI)	p value	Adjusted* Geometric mean (95% CI)	p value
**Total microvascular**					
No	6507	3.26 (3.22-3.30)	**<0.001**	3.43 (3.36-3.51)	**<0.001**
Yes	3262	3.54 (3.47-3.61)		3.61 (3.52-3.71)	
**Nephropathy**					
No	7241	3.31 (3.26-3.35)	**<0.001**	3.48 (3.41-3.56)	0.25
Yes	2501	3.48 (3.41-3.56)		3.54 (3.44-3.64)	
**Retinopathy**					
No	8955	3.34 (3.30-3.38)	**0.010**	3.49 (3.42-3.57)	0.79
Yes	814	3.52 (3.39-3.65)		3.51 (3.37-3.67)	
**Neuropathy**					
No	9204	3.32 (3.28-3.36)	**<0.001**	3.46 (3.39-3.53)	**<0.001**
Yes	558	3.91 (3.69-4.14)		3.99 (3.80-4.19)	
**Microvascular amputation**					
No	9751	3.35 (3.31-3.39)	0.87	3.50 (3.43-3.57)	0.99
Yes	18	3.42 (2.93-4.00)		3.49 (2.69-4.53)	

Geometric means (95% CI) have been reported

*In the adjusted model, geometric means and p values were adjusted for age, sex, treatment allocation, known diabetes duration, smoking (never, former and current), BMI, HbA_{1c}, HOMA-IR, systolic blood pressure, HDL-cholesterol, LDL-cholesterol, triglycerides, fibrinogen, plasma creatinine, homocysteine and glucose-lowering medication (diet alone, oral agent alone, insulin alone and insulin+oral agent) at baseline

Supplementary Table 3: Association of baseline LMW-F levels with on-study CVD events over five years

**Outcome**	**Event (%)**	**HR (95% CI)** (Unadjusted)	Model 1[a]	Model 2[b]
**Total CVD events in all participants**				
Tertile 1	382 (11.7%)	1.00	1.00	1.00
Tertile 2	433 (13.3%)	1.13 (0.99, 1.30)	1.03 (0.90, 1.19)	1.01 (0.87, 1.17)
Tertile 3	476 (14.6%)	1.27 (1.11, 1.46)	1.03 (0.90, 1.18)	0.98 (0.85, 1.13)
Overall effect p value		**0.002**	0.87	0.90
Trend test p value		**<0.001**	0.67	0.74
**Total CVD events in participants with prior CVD**				
Tertile 1	127 (22.4%)	1.00	1.00	1.00
Tertile 2	175 (25.6%)	1.18 (0.94, 1.48)	1.12 (0.89, 1.40)	1.09 (0.86, 1.39)
Tertile 3	236 (27.0%)	1.29 (1.04, 1.60)	1.17 (0.94, 1.45)	1.09 (0.86, 1.37)
Overall effect p value		0.07	0.38	0.72
Trend test p value		**0.02**	0.17	0.49
**Total CVD events in participants without prior CVD**				
Tertile 1	255 (9.5%)	1.00	1.00	1.00
Tertile 2	258 (10.0%)	1.05 (0.88, 1.25)	0.99 (0.84, 1.18)	0.97 (0.81, 1.16)
Tertile 3	240 (10.1%)	1.06 (0.89, 1.27)	0.95 (0.79, 1.13)	0.91 (0.75, 1.09)
Overall effect p value		0.78	0.81	0.58
Trend test p value		0.51	0.54	0.30
**CHD event**				
Tertile 1	150 (4.6%)	1.00	1.00	1.00
Tertile 2	187 (5.7%)	1.24 (1.00, 1.54)	1.12 (0.90, 1.39)	1.07 (0.85, 1.34)
Tertile 3	204 (6.3%)	1.38 (1.11, 1.70)	1.09 (0.88, 1.35)	1.04 (0.83, 1.30)
Overall effect p value		**0.01**	0.57	0.84
Trend test p value		**0.003**	0.46	0.76
**Total stroke**				
Tertile 1	89 (2.7%)	1.00	1.00	1.00
Tertile 2	119 (3.7%)	1.33 (1.01, 1.75)	1.20 (0.91, 1.59)	1.11 (0.83, 1.48)
Tertile 3	124 (3.8%)	1.42 (1.08, 1.86)	1.12 (0.85, 1.47)	1.03 (0.77, 1.37)
Overall effect p value		**0.03**	0.42	0.74
Trend test p value		**0.02**	0.48	0.91
**CVD mortality**				

Tertile 1	64 (2.0%)	1.00	1.00	1.00
Tertile 2	84 (2.6%)	1.29 (0.93, 1.79)	1.13 (0.81, 1.56)	1.04 (0.74, 1.47)
Tertile 3	119 (3.7%)	1.87 (1.38, 2.54)	1.38 (1.01, 1.87)	1.24 (0.90, 1.71)
Overall effect p value		**<0.001**	0.10	0.33
Trend test p value		**<0.001**	**0.04**	0.17
**Coronary or carotid revascularisation**				
Tertile 1	224 (6.9%)	1.00	1.00	1.00
Tertile 2	221 (6.8%)	0.98 (0.81, 1.18)	0.90 (0.75, 1.09)	0.92 (0.76, 1.12)
Tertile 3	251 (7.7%)	1.14 (0.95, 1.37)	0.95 (0.79, 1.14)	0.95 (0.78, 1.15)
Overall effect p value		0.20	0.55	0.70
Trend test p value		0.13	0.68	0.63
**Hospitalisation for angina pectoris**				
Tertile 1	126 (3.9%)	1.00	1.00	1.00
Tertile 2	157 (4.8%)	1.25 (0.98, 1.57)	1.15 (0.91, 1.46)	1.15 (0.90, 1.47)
Tertile 3	175 (5.4%)	1.41 (1.13, 1.78)	1.17 (0.93, 1.48)	1.13 (0.88, 1.44)
Overall effect p value		**0.01**	0.35	0.51
Trend test p value		**0.003**	0.19	0.37

For all the outcomes, tertile 1 was the reference group used for comparison

[a]Model 1 adjusted for age, prior CVD and plasma creatinine

[b]Model 2 adjusted for model 1 + treatment allocation, sex, known diabetes duration, smoking (never, former and current), BMI, HbA1c, HOMA2-IR, systolic blood pressure, HDL cholesterol, LDL cholesterol, triglycerides, fibrinogen, plasma creatinine, homocysteine and glucose-lowering medication (diet alone, oral agent alone, insulin alone and insulin+oral agent) at baseline

Supplementary Table 4: Association of baseline LMW-F levels with on-study microvascular events over five years

Outcome	Event (%)	OR/HR (95% CI) (Unadjusted)	Model 1[a]	Model 2[b]
**Total microvascular disease in all participants**				
Tertile 1	744 (22.9%)	1.00	1.00	1.00
Tertile 2	841 (25.8%)	1.18 (1.05, 1.32)	1.10 (0.98, 1.24)	1.09 (0.96, 1.23)
Tertile 3	879 (27.0%)	1.25 (1.12, 1.40)	1.08 (0.96, 1.21)	1.08 (0.95, 1.21)
Overall effect p value		**<0.001**	0.26	0.35
Trend p value		**<0.001**	0.27	0.28
**Total microvascular disease in participants with prior microvascular disease**				
Tertile 1	253 (25.5%)	1.00	1.00	1.00
Tertile 2	316 (30.7%)	1.30 (1.07, 1.58)	1.23 (1.00, 1.51)	1.21 (0.98, 1.49)
Tertile 3	365 (29.5%)	1.22 (1.01, 1.48)	1.11 (0.91, 1.35)	1.12 (0.91, 1.37)
Overall effect p value		**0.02**	0.14	0.21
Trend p value		0.05	0.40	0.34
**Total microvascular disease in participants without prior microvascular disease**				
Tertile 1	491 (21.7%)	1.00	1.00	1.00
Tertile 2	525 (23.6%)	1.11 (0.97, 1.28)	1.03 (0.89, 1.20)	1.02 (0.87, 1.18)
Tertile 3	514 (25.5%)	1.23 (1.07, 1.42)	1.08 (0.93, 1.26)	1.08 (0.93, 1.26)
Overall effect p value		**0.02**	0.57	0.56
Trend p value		**0.004**	0.29	0.30
**Nephropathy†**				
Tertile 1	493 (16.6%)	1.00	1.00	1.00
Tertile 2	588 (19.7%)	1.23 (1.08, 1.41)	1.15 (1.00, 1.31)	1.13 (0.98, 1.30)
Tertile 3	598 (20.5%)	1.30 (1.14, 1.49)	1.12 (0.98, 1.29)	1.10 (0.96, 1.27)
Overall effect p value		**<0.001**	0.12	0.20
Trend p value		**<0.001**	0.13	0.22
**Neuropathy††**				
Tertile 1	212 (7.1%)	1.00	1.00	1.00
Tertile 2	223 (7.5%)	1.07 (0.88, 1.30)	0.98 (0.80, 1.20)	0.96 (0.78, 1.18)
Tertile 3	232 (8.1%)	1.15 (0.95, 1.40)	0.95 (0.78, 1.17)	0.96 (0.78, 1.18)
Overall effect p value		0.37	0.90	0.91
Trend p value		0.16	0.65	0.71
**Retinopathy***				

Tertile 1	121 (3.7%)	1.00	1.00	1.00
Tertile 2	134 (4.1%)	1.11 (0.87, 1.42)	1.00 (0.77, 1.28)	1.00 (0.77, 1.30)
Tertile 3	147 (4.5%)	1.23 (0.97, 1.57)	1.05 (0.82, 1.35)	1.06 (0.82, 1.38)
Overall effect p value		0.23	0.89	0.86
Trend p value		0.09	0.67	0.62
**2-step progression in ETDRS grade**				
Tertile 1	45 (11.1%)	1.00	1.00	1.00
Tertile 2	31 (10.4%)	0.92 (0.57, 1.50)	0.81 (0.49, 1.36)	0.83 (0.49, 1.43)
Tertile 3	27 (11.4%)	1.03 (0.62, 1.70)	1.00 (0.58, 1.70)	0.97 (0.56, 1.70)
Overall effect p value		0.92	0.69	0.79
Trend p value		0.89	0.95	0.98
**Microvascular amputation***				
Tertile 1	16 (0.5%)	1.00	1.00	1.00
Tertile 2	14 (0.4%)	0.86 (0.42, 1.77)	0.74 (0.36, 1.53)	0.65 (0.31, 1.39)
Tertile 3	22 (0.7%)	1.38 (0.73, 2.64)	1.04 (0.54, 2.01)	1.00 (0.51, 1.96)
Overall effect p value		0.35	0.59	0.44
Trend p value		0.28	0.82	0.89

For all the outcomes, tertile 1 was the reference group used for comparison

*Hazard Ratios are presented for retinopathy and microvascular amputation.

†Patients with macro-albuminuria at baseline were excluded for new/progression of nephropathy. ††Patients with neuropathy at baseline were excluded for new neuropathy.

[a]Model 1 adjusted for age, prior CVD, diabetes duration, smoking (never, former and current), treatment allocation, HbA1c, homocysteine and systolic BP.

[b]Model 2 adjusted for model 1 plus sex, BMI, HOMA2-IR, systolic BP, HDL, LDL, Triglycerides, fibrinogen, and glucose-lowering medication (diet alone, oral agent alone, insulin alone and insulin+oral agent).

Supplementary Table 5: Odds ratio for LMW-F increase/decrease during run-in in response to baseline variables.

	**Unadjusted** Odds ratio	p	**Exhaustive search** Odds ratio	p
**General Characteristics**				
Baseline LMW-F	0.73 (0.69, 0.77)	**<0.001**	0.73 (0.69, 0.77)	**<0.001**
Age	0.99 (0.95, 1.04)	0.77		
Male	0.96 (0.87, 1.06)	0.42		
Caucasian	0.93 (0.77, 1.13)	0.45		
Diabetes duration	0.97 (0.92, 1.02)	0.19		
HbA1c	0.98 (0.94, 1.03)	0.49		
HOMA2-IR	0.95 (0.91, 1.00)	**0.04**	0.93 (0.88, 0.98)	**0.005**
BMI	1.00 (0.95, 1.05)	0.91		
Waist-to-hip ratio	1.00 (0.95, 1.05)	**0.97**		
Systolic BP	1.12 (1.07, 1.18)	**<0.001**	1.13 (1.07, 1.19)	**<0.001**
Diastolic BP	1.08 (1.03, 1.13)	**0.002**		
Pulse pressure	1.09 (1.04, 1.15)	**<0.001**		
Smoking	1.00 (0.94, 1.03)	0.99		
**Renal Function**				
Plasma creatinine	1.03 (0.98, 1.08)	0.27		
eGFR	0.96 (0.92, 1.01)	0.12		
Urine albumin-creatinine ratio	0.96 (0.92, 1.01)	0.09	0.95 (0.90, 0.99)	**0.03**
Cystatin C	0.96 (0.92, 1.01)	0.13		
Uric acid	1.20 (1.14, 1.26)	**<0.001**	1.22 (1.15, 1.28)	**<0.001**
Homocysteine	1.07 (1.01, 1.13)	**0.02**		
**Lipids**				
Total cholesterol	1.04 (0.99, 1.09)	0.13		
HDL-cholesterol	1.00 (0.95, 1.05)	0.89		
LDL-cholesterol	1.03 (0.98, 1.08)	0.26		
Triglycerides	1.03 (0.98, 1.08)	0.29		
**Glucose-lowering medications**				
Diet only	0.98 (0.88, 1.09)	0.68		
Any oral agent	1.10 (0.99, 1.21)	0.06		
Insulin alone	0.77 (0.64, 0.93)	**0.008**		
Insulin + other	0.97 (0.81, 1.17)	0.75		
**Novel Biomarkers**				
White cell count	1.00 (0.95, 1.05)	0.93		
hs-CRP	0.96 (0.92, 1.01)	0.13		
sVCAM-1‡	0.92 (0.88, 0.96)	**<0.001**	0.95 (0.90, 1.00)	**0.04**
sICAM‡	0.95 (0.90, 0.99)	**0.03**		
se-selectin‡	0.99 (0.95, 1.04)	0.84		
IL-6‡	0.95 (0.90, 0.99)	**0.02**		
Fibrinogen	0.98 (0.94, 1.03)	0.53		
Myeloperoxidase‡	0.89 (0.84, 0.93)	**<0.001**	0.88 (0.84, 0.93)	**<0.001**
Leptin‡	1.02 (0.97, 1.07)	0.44		
ox-LDL	1.03 (0.98, 1.09)	0.20		
ox-LDL/LDL	1.05 (1.00, 1.10)	0.07		
**Self-reported clinical history**				
History of hypertension	1.00 (0.91, 1.11)	0.94		
Prior CVD	0.86 (0.77, 0.97)	**0.01**		
Myocardial infarction	0.87 (0.70, 1.08)	0.22		
Stroke	1.07 (0.81, 1.39)	0.64		
Angina	0.82 (0.71, 0.95)	**0.008**		
Peripheral vascular disease	0.90 (0.75, 1.07)	0.24		
Coronary revascularisation (CABG	0.81 (0.63, 1.03)	0.08		

or PTCA)		
Prior microvascular disease	0.92 (0.83, 1.02)	0.10
Retinopathy	0.90 (0.76, 1.07)	0.25
Neuropathy	0.81 (0.63, 1.03)	0.37
Nephropathy	0.93 (0.83, 1.02)	0.22

*****Prior peripheral vascular disease or prior peripheral revascularisation

‡The values below the lower limit of detection were imputed as half the smallest value.

Due to correlation between Cystatin C and eGFR (r=-0.59), eGFR and ox-LDL were not included in multivariable analysis.

Supplementary Table 6: Baseline characteristics of patients who have all three time-points for LMW-F (V1, V4 and V7) versus those who have only two time-points (V1, V4)

	**Subjects with 3 timepoints (n=1988)**	**Subjects with 2 timepoints (n=7725)**	**p value**
**General characteristics**			
LMW-F†	2.56 ± 1.74	3.60 ± 1.71	**<0.001**
Age at visit 1 (years)	62.4 ± 6.8	62.2 ± 6.9	0.15
Male	1238 (62%)	4853 (63%)	0.65
Caucasian	1818 (91%)	7201 (93%)	**0.006**
Diabetes duration† (years)	4.1 ± 2.8	4.4 ± 2.8	**0.02**
HbA1c† (%)	6.8 ± 1.2	7.0 ± 1.2	**<0.001**
HOMA2-IR†	1.7 ± 1.8	1.8 ± 1.8	**0.003**
BMI† (kg/m2)	30.3 ± 1.2	30.2 ± 1.2	0.62
Waist-to-hip ratio†	0.9 ± 1.1	0.9 ± 1.1	0.34
Systolic BP (mmHg)	140 ± 15	141 ± 15	**0.005**
Diastolic BP (mmHg)	82 ± 8	82 ± 9	**0.01**
Pulse pressure (mmHg)	58.0 ± 12.2	58.6 ± 12.5	0.08
Fibrinogen (g/L)	3.5 ± 0.7	3.6 ± 0.7	**<0.001**
Smoking			0.12
Ex-smoker/never	1818 (91%)	6977 (90%)	
Current smoker	170 (9%)	748 (10%)	
**Renal function**			
Plasma creatinine (µmol/L)	76.9 ± 16.4	77.8 ± 15.6	**0.03**
eGFR (mL/min/1.73 m^2)	85.1 ± 14.4	84.3 ± 14.1	**0.03**
Urine albumin-creatinine ratio (mg/mmol)	1.6 ± 3.8	1.5 ± 3.7	0.65
Cystatin C	0.99 ± 0.23	0.92 ± 0.22	**<0.001**
Uric acid (mmol/L)	0.32 ± 1.27	0.32 ± 1.27	**0.002**
**Lipids**			
Total cholesterol (mmol/L)	5.06 ± 0.69	5.03 ± 0.71	0.09
HDL-cholesterol (mmol/L)	1.08 ± 0.26	1.10 ± 0.26	**0.02**
LDL-cholesterol (mmol/L)	3.10 ± 0.64	3.06 ± 0.65	**0.01**
Triglycerides (mmol/L)†	1.78 ± 1.51	1.78 ± 1.49	0.98
**Glucose lowering medication**			**<0.001**
Diet only	521 (26%)	2063 (27%)	
Any oral agent	1257 (63%)	4531 (59%)	
Insulin alone	111 (6%)	494 (6%)	
Insulin other	99 (5%)	637 (8%)	
**Novel biomarkers**			
Homocysteine† (µmol/L)	9.5 ± 1.3	9.7 ± 1.3	**0.005**
White cell count† ($x10^9$/L)	6.6 ± 1.3	6.6 ± 1.3	0.84
hs-CRP† (mg/L)	2.9 ± 3.1	2.7 ± 3.0	0.03
sVCAM-1† (ng/mL)	680.6 ± 1.4	622.0 ± 1.4	**<0.001**
sICAM†‡ (ng/mL)	256.8 ± 1.3	247.9 ± 1.3	**<0.001**
sE-selection†‡ (ng/mL)	35.2 ± 1.5	31.9 ± 1.6	**<0.001**
IL-6†‡ (pg/mL)	2.3 ± 1.9	2.6 ± 1.9	**<0.001**
Myeloperoxidase†‡ (µg/L)	60.0 ± 1.9	48.5 ± 1.9	**<0.001**
Leptin† (pg/mL)	8296 ± 4	8278 ± 3	0.94
ox-LDL/LDL† (mU/mmol)	14.4 ± 1.4	13.0 ± 1.7	**<0.001**

ox-LDL[†] (mU/L)	43.7 ± 1.4	38.7 ± 1.7	**<0.001**
**Clinical history**			
Neuropathy	90 (5%)	465 (6%)	**0.01**

[†]log-transformed, geometric means and geometric SD factors presented.

[‡]The values below the lower limit of detection were imputed as half the smallest value.

T-test for continuous variables, and Chi-square test for categorical variables.

Supplementary Table 7: Treatment effect by LMW-F change during trial run-in

	LMW-F change tertile	Events		HR/OR (95% CI)	P-value	Trend p-value	Interaction p-value
		Placebo (n=4858)	Fenofibrate (n=4855)				
Total CVD events	<0.30 (n=1619)	204 (12.6%)	204 (12.6%)	1.00 (0.82, 1.21)	1.00	0.30	0.31
	0.30-0.98 (n=1658)	226 (13.6%)	177 (11.2%)	0.81 (0.67, 0.99)	**0.036**		
	≥0.98 (n=1581)	246 (15.6%)	227 (13.7%)	0.87 (0.73, 1.04)	0.13		
Total microvascular events	<0.30 (n=1619)	439 (27.1%)	341 (21.1%)	0.72 (0.61, 0.84)	**<0.001**	0.89	0.64
	0.30-0.98 (n=1658)	433 (26.1%)	347 (21.9%)	0.80 (0.68, 0.94)	**0.006**		
	≥0.98 (n=1581)	484 (30.6%)	403 (24.3%)	0.73 (0.62, 0.85)	**<0.001**		

Change defined as Visit 4 - baseline.
HR for Total CVD events and OR for on-study microvascular events presented.

Supplementary Figure 1: Distribution of baseline plasma LMW-F

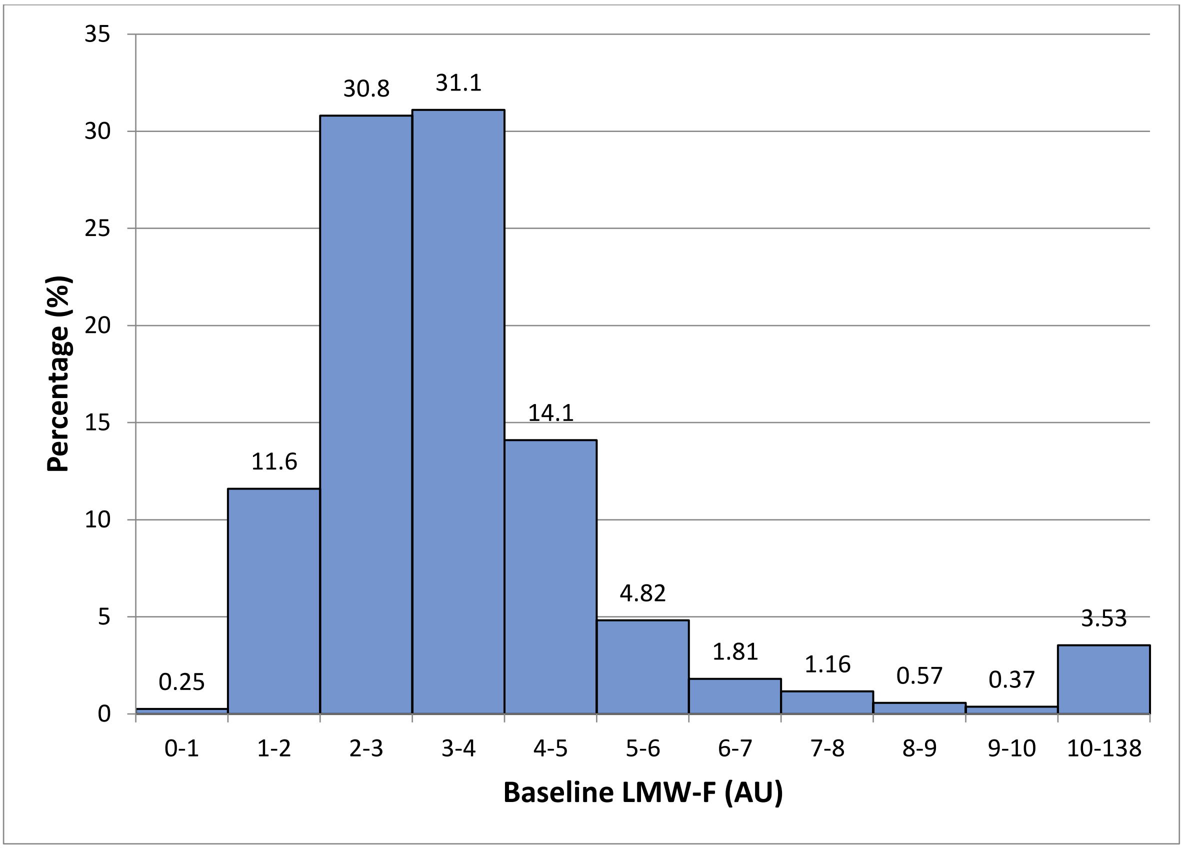

Supplement: Supplementary file 1 — Supplementary Information. [file 41598_2021_98064_MOESM1_ESM.pdf]
